# Proteomic Analysis Identifies ATE1-Dependent Arginylation Dysregulation across Meningioma Grades

**DOI:** 10.1021/acs.jproteome.5c01173

**Published:** 2026-06-29

**Authors:** Janaina Macedo-da-Silva, Benedito Jamilson Araújo Pereira, Simon Ngao Mule, Sueli Mieko Oba-Shinjo, Livia Rosa-Fernandes, Suely K. N. Marie, Giuseppe Palmisano

**Affiliations:** † GlycoProteomics Laboratory, Department of Parasitology, ICB, 54544 University of São Paulo, Sao Paulo CEP: 05508-900, Brazil; ‡ Laboratory of Molecular and Cellular Biology (LIM 15), Department of Neurology, Faculdade de Medicina FMUSP, Universidade de São Paulo, São Paulo CEP: 01246-903, Brazil; § Motor Neuron Disease Research Centre, Faculty of Medicine, Health and Human Sciences, Macquarie Medical School, Sydney, New South Wales 2109, Australia

**Keywords:** arginylation, ATE1, meningiomas, grade comparison, proteomics, post-translational modifications (PTMs)

## Abstract

Meningiomas are the most common primary brain tumors, yet the molecular pathways that distinguish grade 1 from grade 2 lesions remain insufficiently understood. Among post-translational modifications, N-terminal arginylation—catalyzed by ATE1—regulates protein stability and cellular stress responses, but its role in meningioma biology has not been explored. Here, we integrated mass-spectrometry–based proteomics, immunoblotting, and transcriptomic reanalysis to investigate pathway regulation across tumor grades. Grade 1 meningiomas displayed higher ATE1 expression and increased arginylation of key chaperones, accompanied by activation of the PERK branch of the unfolded protein response (UPR), enhanced autophagy, and greater engagement of apoptotics pathways. In contrast, grade 2 tumors showed reduced ATE1 levels, diminished BIP arginylation, attenuated UPR-PERK signaling, impaired autophagy, and increased proliferative signaling. Proteins predicted to be substrates of ATE1-mediated degradation were upregulated in grade 2 tumors, suggesting that loss of arginylation may stabilize pro-oncogenic factors. Together, these findings reveal grade-specific remodeling of the N-degron/arginylation axis and highlight protein arginylation as a previously unrecognized modulator of meningioma progression, with potential therapeutic relevance.

## Graphical abstract

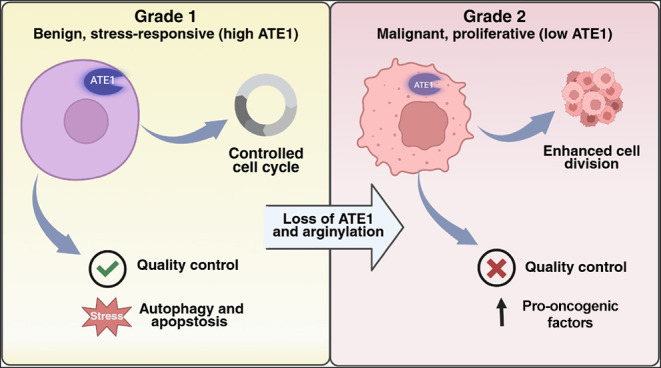

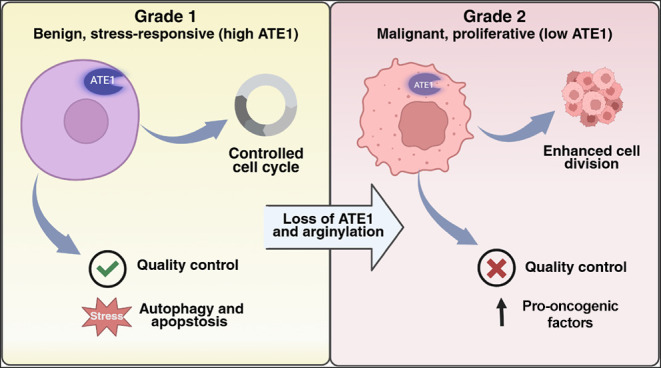

## Introduction

Meningiomas are the most common primary tumors of the central nervous system, arising from the meningeal layers that envelop the brain and spinal cord.^1^ The World Health Organization (WHO) classifies them into grades 1 (G1), 2 (G2), and 3 (G3) based on histopathological and molecular features.^2^ G1 meningiomas, which represent approximately 81% of cases, are typically benign and slow-growing, with recurrence rates around 5%. G2 tumors account for roughly 18% of cases and display higher mitotic activity, increased aggressiveness, and recurrence rates of up to 40% following surgical resection; their ten-year survival ranges from 50% to 79%. G3 meningiomas, although rare (1–5%), exhibit marked anaplasia, necrosis, brain invasion, and high mitotic indices, and are associated with poor ten-year survival (14–24%).^3,4^

Therapeutic decision-making for meningioma is strongly guided by tumor grade.^5,6^ However, noninvasive imaging modalities lack the resolution to distinguish grades reliably,^7^ and only about 40% of cases are histologically confirmed, as diagnosis typically requires surgical intervention.^8^ As in other neuro-oncological contexts, molecular characterization is therefore essential to support diagnosis, refine tumor classification, and improve clinical management, particularly for the prevalent G1 and G2 tumors.^9^

Genomic studies have identified recurrent alterations in *NF2*, *SMO*, *TRAF7*, *AKT1*, *PIK3CA*, *KLF4*, and *TERT* promoter genes,^10,11^ yet comprehensive insights into pathway regulation, proteomic signatures, and post-translational modifications (PTMs) in meningiomas remain limited.^12^ A better understanding of the molecular mechanisms underlying tumor initiation and progression is necessary to advance therapeutic strategies.^13^

Among PTMs, N-terminal arginylation has emerged as a key regulator of cell migration, adhesion, proliferation, and proteostasis in several cancers, including prostate cancer,^14^ melanoma,^15^ and glioblastoma.^16^ This PTM is mediated by arginyltransferase 1 (ATE1), which transfers an arginine residue to N-terminal glutamic acid, aspartic acid, or oxidized cysteine residues, generating destabilizing N-degrons that target proteins for degradation via proteasomal and autophagy pathways. Although dysregulation of extracellular matrix remodeling, chromatin dynamics, RNA metabolism, and canonical oncogenic pathways (e.g., TNF-α, c-Myc) has been reported in meningiomas,^17^ the contribution of the N-degron/arginylation system to meningioma biology has not yet been explored.

To address this gap, we applied mass-spectrometry–based proteomics to G1 (*n* = 7) and G2 (*n* = 5) meningioma samples and complemented these data with immunoblotting to interrogate the N-degron/arginylation pathway, unfolded protein response (UPR), autophagy, cell death, and proliferation mechanisms. Findings were further validated using an independent public transcriptomic cohort.^18^ We identified upregulation of ATE1 in G1 tumors, consistent with enhanced arginylation activity and its known association with BIP-mediated UPR activation. Immunoblotting confirmed selective activation of the PERK branch of the UPR in G1 tumors, while the IRE1A branch was downregulated. Downstream processes, including p62/SQSTM1-dependent autophagy, mTOR-regulated autophagy induction, and cell death pathways, were also enriched in G1 samples. In contrast, proliferative signaling pathways predominated in G2 tumors. Together, these data reveal extensive pathway remodeling between meningioma grades and identify attenuation of the arginylation pathway in G2 tumors as a previously unrecognized feature of meningioma progression.

## Materials and Methods

### Patients

This study was approved by the Ethics Committee of the University of São Paulo under number 830/01. Written informed consent was obtained from all participants. Tumor specimens were collected by neurosurgeons from the clinical staff of Hospital das Clínicas of the University of São Paulo. All collected materials were aliquoted with sterile material and stored at −80 °C. Histological characterization was conducted, and all patients were diagnosed with G1 (*n* = 7) or G2 (*n* = 5) meningiomas, as presented in Table 1.

**Table 1:** Details of Patients Included in This Study: Diagnosis, Recurrence Status, Gender, Age, and Localization

Diagnostic	Recurrence	Gender	Age	Localization
G1 (Transitional)	0	F	54	Skull base
G1 (Transitional)	1	F	46	Convexity
G1 (Transitional)	0	M	43	Convexity
G1 (Transitional)	0	F	60	Convexity
G1 (Transitional)	0	F	72	Convexity
G1 (Meningothelial)	0	F	51	Skull Base
G1 (Meningothelial)	1	M	58	Convexity
G2	1	M	39	Convexity
G2	1	F	55	Convexity
G2	1	F	64	Convexity
G2	0	F	33	Convexity
G2	0	M	60	Convexity

### Sample Preparation for Mass Spectrometry Analysis

Tissue samples were homogenized in a buffer containing 10 mM HEPES, 1% SDS, 1.5 mM MgCl_2_.6H_2_O, 10 mM KCl, 1 mM DTT, 0.1% NP-40, protease (cOmplete, Sigma-Aldrich), and phosphatase inhibitors (PhosStop, Sigma–Aldrich). The samples were sonicated for 30 s for five cycles, centrifuged at 15,000*g* at 4 °C for 15 minutes, and the supernatant was collected. Protein quantification was performed by fluorometric assay using the Qubit platform, after 100x dilution of the samples. A total of 30 μg of proteins was solubilized in Laemmli buffer (Bio-Rad) and boiled at 100 °C for 10 min. The proteins were then loaded onto a 12% SDS gel and electrophoresed for 15 min at 130 V until they entered the gel. After staining with Coomassie Blue, the bands were excised, agitated with 50 mM ammonium bicarbonate (Ambic) in 40% acetonitrile (ACN), and dehydrated with 100% ACN. Samples were then reduced with 10 mM dithiothreitol (DTT) at 56 °C for 45 min and alkylated with 55 mM iodoacetamide (IAA) for 30 min at room temperature in the absence of light. The samples were subsequently digested for 16 h with trypsin (Promega) at a ratio of 1:50 (enzyme/substrate) at 37 °C. After digestion, all the samples were acidified with 10% (v/v) trifluoroacetic acid (0.1% v/v final concentration) to terminate proteolysis. Finally, samples were desalted using C18 internal stage tips (3M Empore), dried in a SpeedVac, and resuspended in 0.1% formic acid (FA) for mass spectrometry analysis.

### Mass Spectrometry Analysis

Mass spectrometry analyses were performed utilizing an Easy NanoLC 2 (Thermo Scientific) coupled online to LTQ-Orbitrap Velos (Thermo Scientific). The digested peptides were loaded onto an EASY C18 trap column (100 μm id × 2 cm, 5 μm particle size, 120 Å pore size) and chromatographically separated using a PepMap C18 column (75 μm id × 10 cm, 3.5 μm particle, 100 Å pore size). The mixture was separated by a gradient from 2 to 30% solvent B for 80 min; 30–95% solvent B for 5 min, and 20 min at 95% solvent B (A = 0.1% FA; B = 95% CAN, 0.1% FA). The instrument was configured for data-dependent acquisition (DDA) and operated in positive mode. Scans (350–1,500 *m*/*z*) were acquired at 60,000 resolution. The 20 most intense ions (excluding the unassigned and 1+ charge states) were isolated and collision-induced dissociation (CID) was performed using a normalized collision energy of 35. General mass spectrometry conditions included a spray voltage of 1.9 kV, no sheath or auxiliary gas flow, a heated capillary temperature of 280 °C, and predictive automatic gain control (AGC).

### Data Analysis

The corresponding raw files were analyzed with MaxQuant software version 1.6.10.4.3,^19^ employing the Andromeda search engine. The *Homo sapiens* Swiss-Prot database was downloaded in December 2023. Trypsin served as the cleavage enzyme (fully tryptic), allowing a maximum of two missed cleavage sites. In the search for tryptic peptides, carbamidomethylation of cysteine and oxidation of methionine were considered static and dynamic modifications, respectively. The false discovery rate (FDR) was set to 1% for peptide and protein identification. Label-free quantification (LFQ) was conducted by applying an extracted-ion chromatogram (XIC) area.

The identified proteins were filtered to obtain a minimum of five valid values for each group. Subsequently, a *t* test (*p* < 0.05) was applied, and differentially regulated proteins were used for Gene Ontology and String network^20^ analysis using the Cytoscape software.^21^ Principal component analysis was performed using proteins quantified in all samples and using the MetaboAnalyst platform.^22^

### RNA-Seq Data Reanalysis

Data from Patel et al.^18^ were downloaded from the Gene Expression Omnibus (GEO) database under accession number GSE136661. Functions for organizing gene annotations, processing, and normalizing data were applied using limma,^23^ Glimma,^24^ edgeR, and Homo.sapiens packages in Rstudio.^25^ Differentially regulated genes were determined by applying a cutoff of adjusted *p* < 0.01 (Benjamini-Hochberg).

### Western Blotting

A total of 20 μg of proteins were separated by SDS-PAGE and electrotransferred onto PVDF membranes, which were subsequently incubated with a blocking buffer containing 5% bovine serum albumin (BSA) in Tris-buffered saline (TBS) and 0.05% Tween- 20 (TBS-T) for 45 min. After three washes with TBS-T, the membranes were incubated overnight at 4 °C with primary antibodies (Table 2). The membranes were washed three times with TBS-T and incubated with the respective secondary antibodies (Table 2) for 1 h at room temperature. For detection of arginylated and total protein levels on the same membrane, sequential immunoblotting with membrane stripping was performed. Membranes were first probed with the antibody recognizing the arginylated form (e.g., anti-R-ACTB). After chemiluminescent detection, membranes were stripped using a mild stripping buffer (25 mM glycine-HCl, 1% SDS, pH 2.0) for 30 min at room temperature with gentle agitation. Stripping efficiency was confirmed by exposing membranes to the secondary antibody alone and detecting no residual signal. Membranes were then reblocked with 5% BSA in TBS-T for 45 min and reprobed with the antibody against the total protein (e.g., anti-ACTB). All signals were normalized to their respective loading controls (tubulin), and ratios of arginylated to total protein were calculated from sequential blots performed on the same membrane. Control experiments to check the stripping efficiency were performed and showed no residual signal after the stripping. All Western blot experiments were repeated at least three independent times with consistent results. Immunoreactive bands were detected using the ChemiDoc Imaging System, and protein quantification was performed using ImageJ software.

**Table 2:** Primary and Secondary Antibodies Used in Western Blotting Analyses, with Their Respective Dilutions, Reference Catalog Numbers, Type, and Supplier Company

Antibody	Secondary Antibody	Dilution	Reference	Type	Company
Anti-ACTB	Goat Anti-Mouse	1:10,000	#A2228	Monoclonal	Sigma-Aldrich
Anti-AKT	Goat Anti-Rabbit	1:1000	#9272	Polyclonal	Cell Signaling
Anti-APAF1	Goat Anti-Rabbit	1:1000	#8969	Monoclonal	Cell Signaling
Anti-ATE1	Goat Anti-Rat	1:1000	MABS436	Monoclonal	Merck Millipore
Anti-ATF4	Goat Anti-Rabbit	1:1000	#11815	Monoclonal	Cell Signaling
Anti-ATG5	Goat Anti-Rabbit	1:1000	#12994	Monoclonal	Cell Signaling
Anti-BAX	Goat Anti-Rabbit	1:1000	#5023	Monoclonal	Cell Signaling
Anti-BCL2	Goat Anti-Rabbit	1:1000	#3498	Monoclonal	Cell Signaling
Anti-BIP	Goat Anti-Rabbit	1:1000	#3183	Polyclonal	Cell Signaling
Anti-CALR	Goat Anti-Rabbit	1:1000	ab2907	Polyclonal	Abcam
Anti-EIF2A	Goat Anti-Rabbit	1:1000	#9722	Monoclonal	Cell Signaling
Anti-IRE1A	Goat Anti-Rabbit	1:1000	#3294	Monoclonal	Cell Signaling
Anti-LC3B	Goat Anti-Rabbit	1:1000	#PA116930	Polyclonal	Thermo
Anti-MLKL	Goat Anti-Rabbit	1:1000	#37705	Monoclonal	Cell Signaling
Anti-mTOR	Goat Anti-Rabbit	1:1000	# 701483	Monoclonal	Thermo
Anti-p38	Goat Anti-Rabbit	1:1000	#9212	Polyclonal	Cell Signaling
Anti-p62	Goat Anti-Rabbit	1:1000	#18420–1AP	Polyclonal	Thermo
Anti-P-AKT	Goat Anti-Rabbit	1:1000	#4060	Monoclonal	Cell Signaling
Anti-P-EIF2A	Goat Anti-Rabbit	1:1000	#9721	Monoclonal	Cell Signaling
Anti-PERK	Goat Anti-Rabbit	1:1000	#3192	Monoclonal	Cell Signaling
Anti-P-IRE1A	Goat Anti-Rabbit	1:1000	ab48187	Polyclonal	Abcam
Anti-P-mTOR	Goat Anti-Rabbit	1:1000	#PA5104898	Polyclonal	Thermo
Anti-PDI	Goat Anti-Mouse	1:1000	# MA3–019	Polyclonal	Thermo
Anti-P-p38	Goat Anti-Rabbit	1:500	#9211	Polyclonal	Cell Signaling
Anti-P-PERK	Goat Anti-Rabbit	1:500	#3179	Monoclonal	Cell Signaling
Anti-R-ACTB	Goat Anti-Mouse	1:1000	ABT264	Polyclonal	Merck Millipore
Anti-R-BIP	Goat Anti-Rabbit	1:1000	ABS2103	Polyclonal	Merck Millipore
Anti-R-CALR	Goat Anti-Rabbit	1:1000	ABS1671	Polyclonal	Merck Millipore
Anti-R-PDI	Goat Anti-Rabbit	1:1000	ABS1655	Polyclonal	Merck Millipore
Anti-TUB	Goat Anti-Mouse	1:10,000	#3873	Monoclonal	Cell Signaling
Anti-Ub	Goat Anti-Rabbit	1:1000	#43124	Monoclonal	Cell Signaling
Anti-XBP1	Goat Anti-Rabbit	1:1000	#12782	Monoclonal	Cell Signaling
Goat Anti-Mouse	-	1:1000	PI-2000–1	Polyclonal	Vector Laboratories
Goat Anti-Rabbit	-	1:1000	PI-1000–1	Polyclonal	Vector Laboratories
Goat Anti-Rat	-	1:1000	BA-9400	Polyclonal	Vector Laboratories

## Results

To investigate pathways differentially regulated between G1 and G2 meningiomas, we integrated mass-spectrometry–based proteomics with immunoblotting analyses and *in silico* evaluation of public omics data sets (Figure S1A). Proteomic comparison of G1 (*n* = 7) and G2 (*n* = 5) tumors revealed clear separation in principal component analysis (PCA), primarily along component 1 (32.8%) (Figure 1A). After filtering to include proteins present in all samples within each group, 81 proteins were identified as differentially regulated, with 58 upregulated and 23 downregulated (Figure 1B). Gene ontology enrichment (Figure 1C) showed that G2 tumors were characterized by increased expression of proteins involved in apoptosis, gene expression, chaperone activity, and ATP metabolism. Conversely, proteins linked to cytoskeleton organization and the annexin family were downregulated in G2 tumors (Table S3).

**Figure 1:**
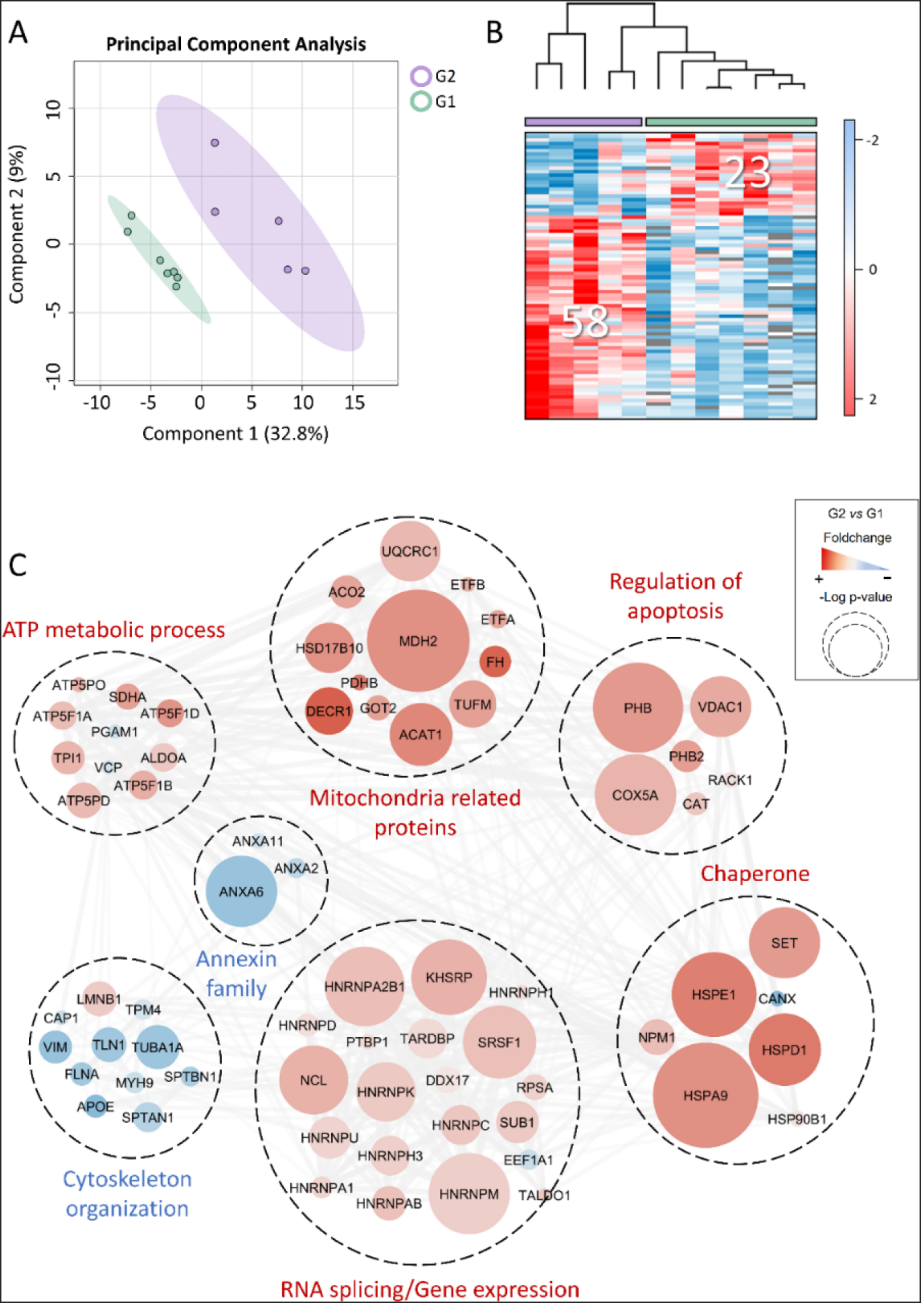
Proteomic profiling of grade 1 and grade 2 meningiomas. (A) Principal component analysis (PCA) of grade 1 (G1, *n* = 7) and grade 2 (G2, *n* = 5) tumors showing group separation. (B) Heatmap of proteins differentially expressed between G1 and G2 tumors (*t* test, *p* < 0.05). Protein intensities were z-score normalized; red and blue indicate higher and lower abundance in G2 relative to G1, respectively. (C) Functional interaction network of upregulated (red) and downregulated (blue) proteins, annotated with enriched ontologies, domains, pathways, and UniProt keywords (*q*-value <0.05, Benjamini-Hochberg). Node size reflects -log­(p-value), and color intensity represents fold-change magnitude.

Given the central role of the N-degron/arginylation pathway, we next assessed the expression of its components. Reanalysis of public transcriptomic data (18) demonstrated significant downregulation of ATE1 and E3 ubiquitin ligases UBR2, UBR4, and UBR5 in G2 tumors (Figure 2A). Consistently, immunoblotting in an independent cohort confirmed reduced ATE1 protein levels in G2 meningiomas (*p* = 0.0056) (Figure 2B and Figure S1B). Arginylation of the ER chaperones CALR (*p* = 0.0403) and PDI (*p* = 0.0287) was also diminished, whereas arginylation of β-actin (ACTB) was increased (*p* = 0.0011) (Figure 2C and Figure S1B). No significant changes in total ubiquitination were detected (Figure 2D). These findings parallel reports of chaperone modulation and ATE1-dependent UPR activation in other tumors, such as glioblastoma.^16^

**Figure 2:**
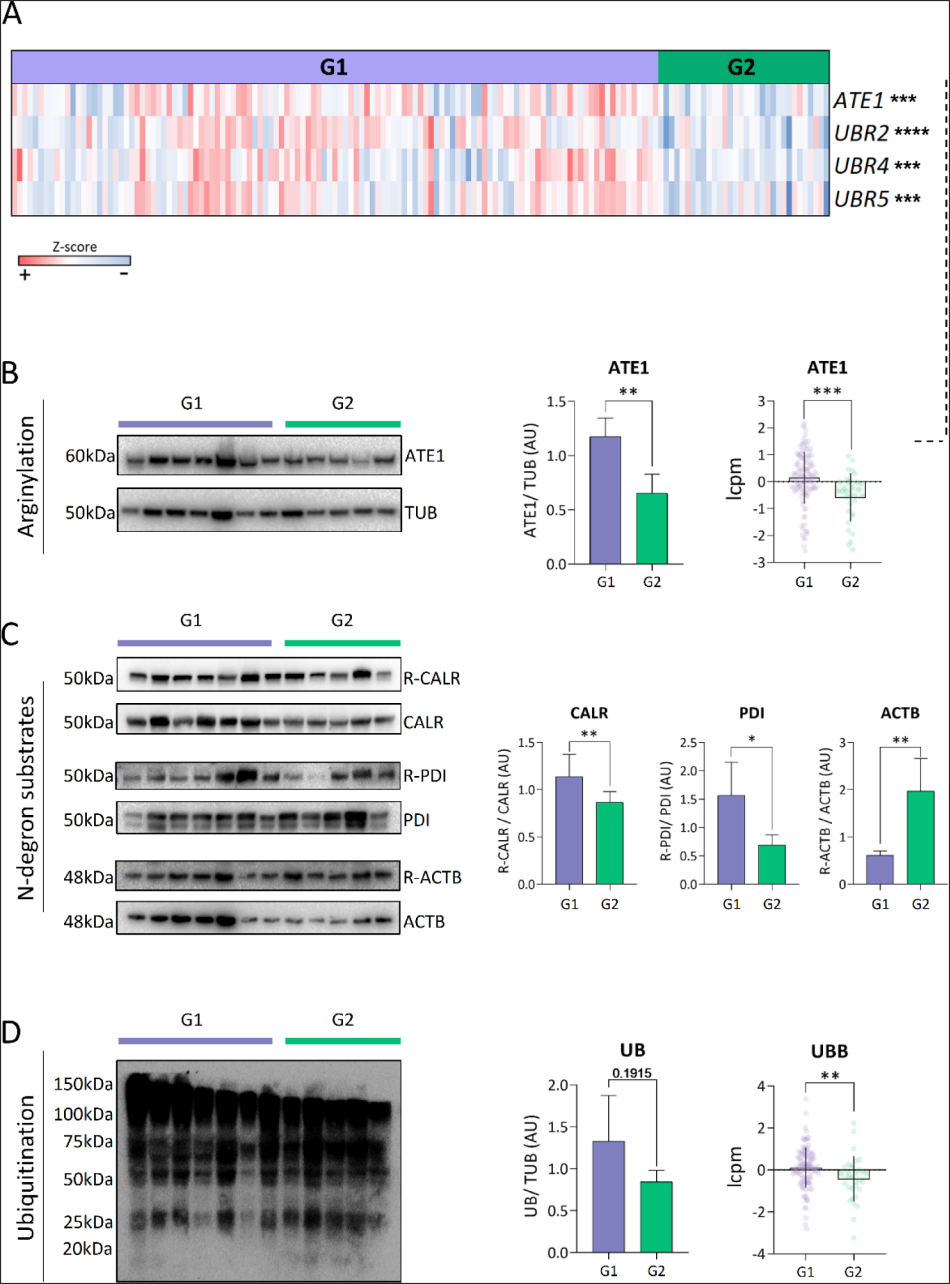
Regulation of the N-degron pathway in grade 1 (G1) and grade 2 (G2) meningiomas. (A) Heatmap showing expression of *ATE1*, *UBR2*, *UBR4*, and *UBR5* in the transcriptomic data set from Patel et al..^18^ LCPM values were *z*-score normalized. Genes were selected using a *t* test (*q* < 0.01, Benjamini-Hochberg, G2 vs G1). Red and blue indicate upregulated and downregulated genes, respectively. (B) Representative immunoblot of ATE1 levels in G1 (*n* = 7) and G2 (*n* = 5) tumors, normalized to tubulin (TUB). Adjacent boxplot shows ATE1 transcript levels from Patel et al. (C) Representative immunoblots of ATE1 substrates, including arginylated BIP, PDI (both bands quantified), and β-actin (ACTB). (D) Representative immunoblots of total protein ubiquitination in G1 and G2 tumors, with corresponding ubiquitin B (*UBB*) transcript levels from Patel et al. ****p < 0.0001; ***p < 0.001; **p < 0.005; *p < 0.05.

To further examine UPR regulation, we analyzed transcriptomic data from Patel et al.,^18^ which revealed distinct expression profiles for genes associated with UPR activation, ER stress, and ER-associated degradation (ERAD) (Figure 3A). PCA using these gene sets showed robust discrimination between G1 and G2 tumors (Figure 3B). In line with reduced ATE1 activity, arginylation of BIP—the primary UPR sensor—was decreased in G2 tumors (Figure 3C and Figure S2). Immunoblotting revealed downregulation of the PERK branch and activation of the IRE1A branch of the UPR in G2 meningiomas (Figure 3C and Figure S2).

**Figure 3:**
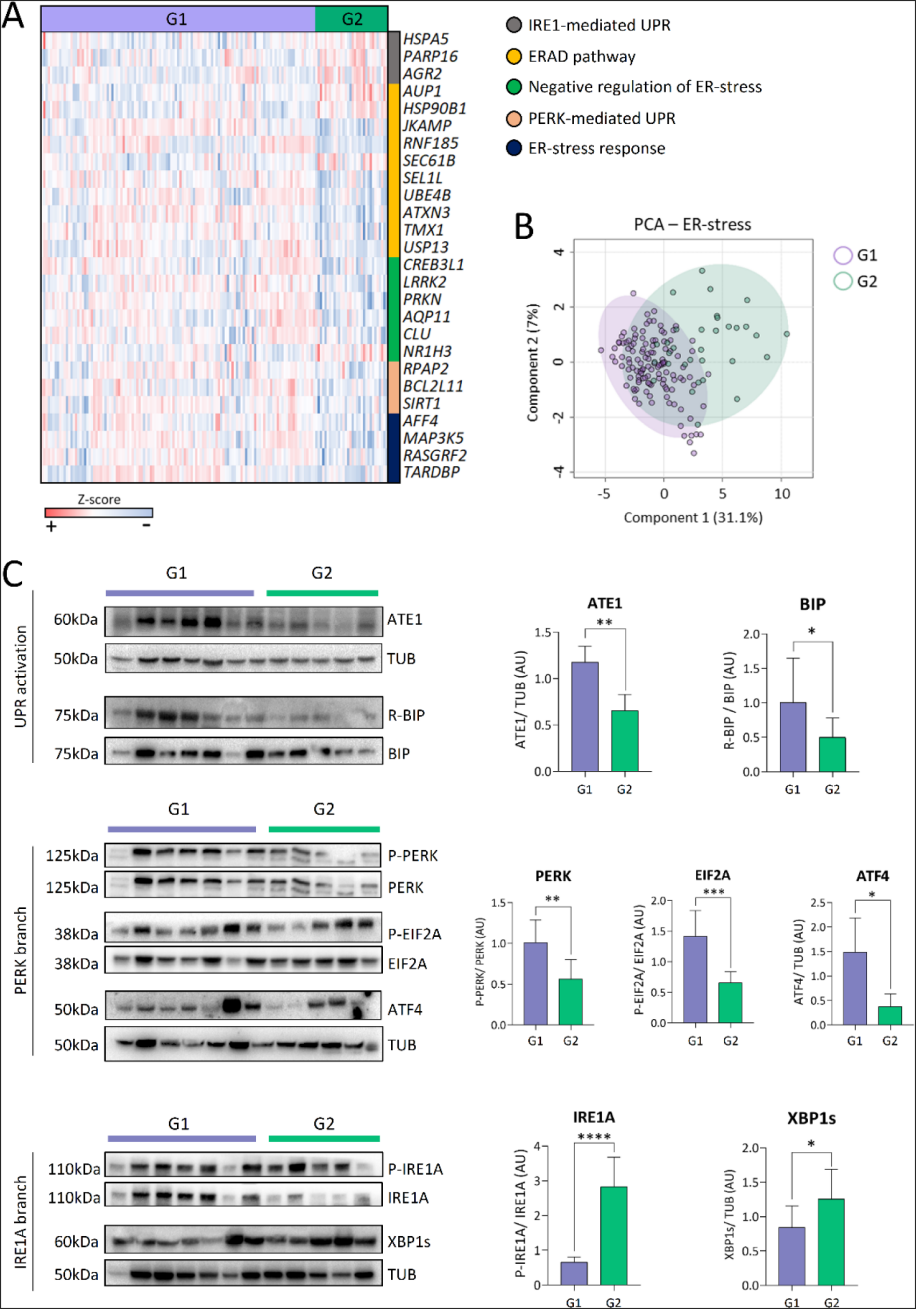
Regulation of unfolded protein response (UPR) pathways in grade 1 (G1) and 2 (G2) meningiomas. (A) Heatmap of genes associated with ER stress, PERK-mediated UPR, endoplasmic-reticulum­(ER)-associated protein degradation (ERAD), negative regulation of ER stress, and IRE1-mediated UPR in the data set of Patel et al..^18^ LCPM values were z-score normalized. Genes were selected using a *t* test (q < 0.01, Benjamini-Hochberg, G2 vs G1). Red and blue indicate higher and lower expression in G2, respectively. (B) Principal component analysis (PCA) using genes annotated to the ER stress response pathway. (C) Representative immunoblots showing UPR pathway components, including ATE1, BIP, and the PERK and IRE1A signaling branches. ****p < 0.0001; ***p < 0.001; **p < 0.005; *p < 0.05.

Because UPR modulation influences autophagy and cell death, we next examined these downstream pathways (Figure 4). The autophagy adaptor p62/SQSTM1 was elevated in G2 tumors, indicating impaired autophagic flux (Figure 4A and Figure S3A). LC3B levels, which reflect autophagosome formation, were reduced in G2 samples (Figure 4C and Figure S3A). mTOR phosphorylation, a key regulator of autophagy induction, was also decreased in G2 tumors, consistent with reduced PERK signaling and diminished ATE1 expression. Markers of apoptosis demonstrated the opposite trend: APAF1, MLKL, and the BAX/BCL2 ratio were higher in G1 tumors, indicating enhanced activation of cell death pathways in this group (Figure 4 and Figure S3B).

**Figure 4:**
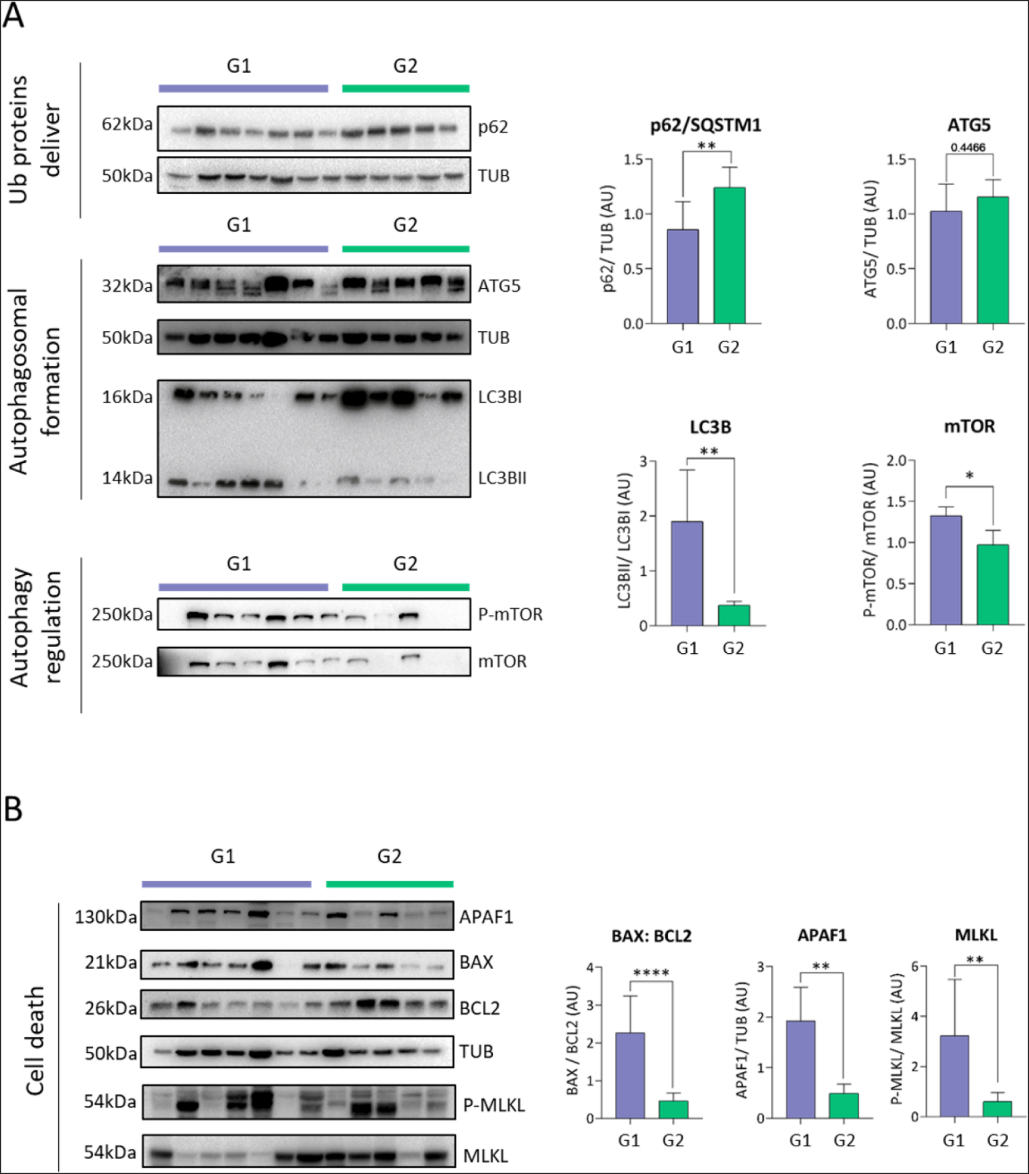
Regulation of autophagy and cell death pathways in grade 1 (G1) andgrade 2 (G2) meningiomas. (A) Representative immunoblots of autophagy markers, including the cargo adaptor p62/SQSTM1, LC3B and ATG5 (autophagosome formation), and phosphorylated mTOR (autophagy regulation). (B) Representative immunoblots of cell death markers APAF1, BAX, BCL2, and MLKL. Both phosphorylated MLKL bands were quantified. ****p < 0.0001; ***p < 0.001; **p < 0.005; *p < 0.05.

Finally, we evaluated proliferative signaling. G2 tumors showed increased expression of transcripts encoding proteins susceptible to N-terminal arginylation and involved in cell cycle progression, cell division, and DNA metabolic processes (Figure 5A). Classical proliferation pathways were also activated: immunoblotting revealed elevated phosphorylation of AKT and p38/MAPK in G2 tumors (Figure 5B and Figure S4). Together, these results demonstrate the coordinated repression of the N-degron/arginylation and PERK-mediated UPR pathways in G2 meningiomas, accompanied by reduced autophagy, diminished cell death signals, and enhanced proliferative activity.

**Figure 5:**
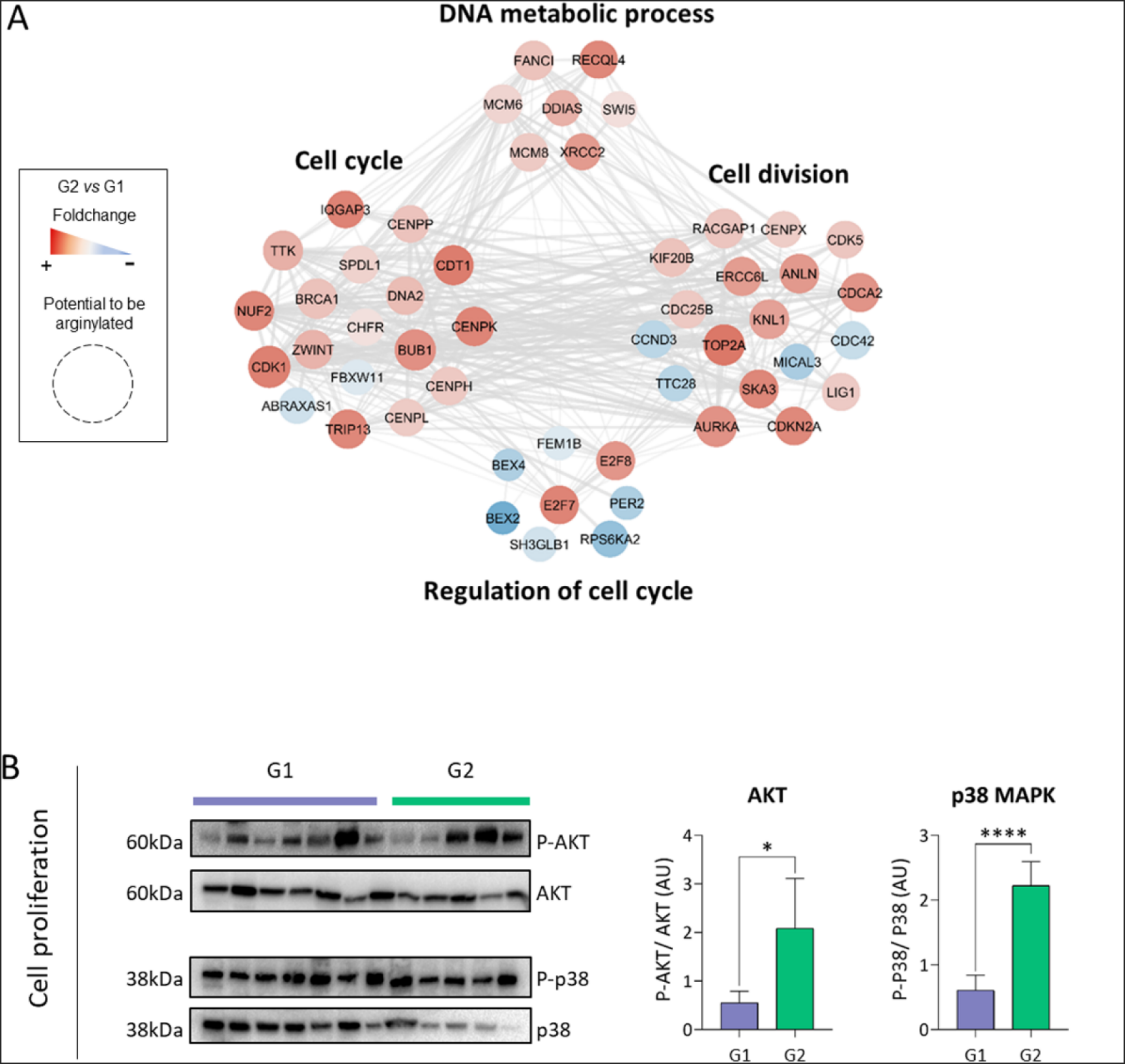
Regulation of proliferation-associated pathways and arginylation-susceptible transcripts in meningiomas. (A) Network representation of transcripts involved in cell cycle regulation, cell division, and DNA metabolic processes that contain N-terminal residues associated with arginylation (oxidized cysteine, glutamic acid, and aspartic acid). Node color reflects the log fold-change (G2 vs G1); only transcripts with *q* < 0.01 (Benjamini-Hochberg) are shown. (B) Representative immunoblots of proliferation markers associated with AKT and p38 MAPK phosphorylation. ****p < 0.0001; *p < 0.05.

## Discussion

Meningiomas are the most common intracranial tumors in adults, with G1 and G2 lesions representing the vast majority of diagnoses.^26,27^ While G1 meningiomas typically follow an indolent clinical course, G2 tumors exhibit higher recurrence rates and greater biological aggressiveness.^28^ Thus, elucidating the molecular determinants that distinguish these grades is essential for improving prognostic assessment and therapeutic strategies. In this study, we integrated label-free proteomics, immunoblotting, and transcriptomic reanalysis to identify pathways differentially modulated across meningioma grades. Our multiomic approach revealed broad alterations in RNA processing, cytoskeletal dynamics, apoptosis, chaperone regulation, and energy metabolism (Figure 1C), consistent with previous proteomic studies.^17^ Importantly, however, our study uncovered previously unrecognized dysregulation of the N-degron/arginylation pathway, positioning protein arginylation as a novel regulator of meningioma progression.

A central finding of this work is the grade-specific regulation of the arginyltransferase ATE1, the enzyme responsible for N-terminal arginylation. ATE1 is a multifunctional regulator that influences protein homeostasis, stress response, cytoskeleton maintenance, and cell migration.^29^ We observed consistent downregulation of ATE1 and associated N-degron components in G2 tumors at both transcript and protein levels. Moreover, arginylation of key chaperones—including BIP, CALR, and PDI—was significantly reduced in G2 meningiomas. These findings mirror our recent observation in glioblastoma, in which decreased BIP arginylation impaired activation of the unfolded protein response (UPR) .^16^ Additional studies have further linked glioblastoma aggressiveness to dysregulation of PERK-mediated UPR signaling^30^ and a mechanoadaptive PERK/FLNA/F-actin axis,^31^ underscoring the importance of ER stress resolution mechanisms in malignant behavior. Our results now extend these concepts to meningiomas by revealing that G1 tumors- characterized by higher ATE1 expression and increased BIP arginylation—preferentially activate the PERK branch of the UPR, whereas G2 tumors show reduced PERK activity and compensatory activation of IRE1A (Figure 3). This shift likely reflects altered stress-adaptation capacity linked to diminished arginylation.

UPR modulation has critical downstream effects on autophagy and apoptosis.^32,33^ In line with enhanced PERK signaling, G1 meningiomas exhibited increased autophagic flux (higher LC3B, lower p62/SQSTM1, elevated mTOR phosphorylation) and upregulation of pro-apoptotic markers including APAF1 and MLKL (Figure 4). In contrast, G2 tumors exhibited impaired autophagy and attenuated apoptosis, a pattern consistent with diminished ATE1-mediated arginylation and diminished stress signaling. Previous work reported minimal differences in apoptotic gene expression between G1 and G2 meningiomas;^34^ but our integrated proteomic and functional pathway analysis reveals a more nuanced regulatory landscape, likely attributable to stress-response mechanisms not captured at the transcript level. These findings align with broader literature linking ER stress and UPR remodeling to tumorigenic processes in prostate,^35^ breast,^36,37^ colorectal,^38^ and pancreatic cancers,^39–41^ reinforcing the relevance of stress-adaptation pathways in cancer progression.

In addition to stress-response pathways, proliferation-associated signaling was markedly elevated in G2 tumors. Increased phosphorylation of AKT and p38/MAPK (Figure 5) is consistent with reports connecting these pathways to more aggressive meningioma phenotypes.^42^ Similarly, ATE1 promoted breast cancer progression via arginylation-dependent regulation of MAPK-MYC signaling.^43^ Notably, many transcripts upregulated in G2 meningiomas encode proteins predicted to be susceptible to arginylation, including regulators of cell-cycle control and DNA metabolism. When considered alongside diminished ATE1 expression, these findings raise the possibility that loss of arginylation may stabilize pro-proliferative substrates, promote invasive behavior,^15^ and inversely correlate with metastasis,^44^ thereby enhancing tumor growth and contributing to the more malignant phenotype.

## Conclusions

Collectively, these findings position protein arginylation as a previously unrecognized and functionally significant modulator of meningioma biology. The coordinated downregulation of ATE1, reduced substrate arginylation, altered UPR branch engagement, impaired autophagy, diminished apoptosis, and heightened proliferative signaling converge on a model in which attenuation of the N-degron/arginylation pathway contributes to the increased aggressiveness of G2 meningiomas. By identifying the arginylation pathway as a key integrator of proteostasis and stress adaptation, this work not only expands the molecular understanding of meningioma progression but also introduces a promising new axis for mechanistic exploration and potential therapeutic intervention.

## Supplementary Material









## Data Availability

Project Name: Proteomic analysis identifies ATE1-dependent arginylation dysregulation across meningioma grades Project Accession: PXD071919 Project DOI: Not applicable

## Notes

Supporting Information: cover page (PDF)
Figure S1: schematic overview of the experimental workflow used in this study. Original immunoblot images corresponding to the quantification shown in Figure 2. Figure S2: original immunoblot images corresponding to the quantification shown in Figure 3. Figure S3: original immunoblot images corresponding to the quantification shown in Figure 4 for autophagy markers. Figure S4: original immunoblot images corresponding to the quantification shown in Figure 5 (PDF)
Table S1: details of patients included in this study: diagnosis, recurrence status, gender, age, localization, follow-up, death, loss of Chr.22, and NF2 status. Table S2: differentially regulated proteins between the comparison of grade (G1) and grade 2 (G2) groups (*p*-value <0.05). Table S3: analysis of gene ontology, domains, and enriched pathways (FDR < 0.05). Table S4: Limma output file on the transcriptomics data provided by Patel et al. (PMID: 31591222) (XLSX)
Table S5: protein groups (TXT)
