# Supplementary material for: Proteomic Analysis Identifies ATE1-Dependent Arginylation Dysregulation across Meningioma Grades
Source: J Proteome Res. 2026 Jun 29;25(8):3926–36. doi: 10.1021/acs.jproteome.5c01173 (PMC13459535; doi:10.1021/acs.jproteome.5c01173)
Supplement: Supplementary file 1 [file pr5c01173_si_001.pdf]

Proteomic analysis identifies ATE1-dependent arginylation dysregulation across meningioma grades

Janaina Macedo-da-Silva[1], Benedito Jamilson Araújo Pereira[2], Sueli Mieko Oba Shinjo[2], Livia Rosa-Fernandes[3], Suely N. K. Marie[2*], Giuseppe Palmisano[1*]

1. GlycoProteomics Laboratory, Department of Parasitology, ICB, University of São Paulo, Sao Paulo, Brazil

2. Laboratory of Molecular and Cellular Biology (LIM 15), Department of Neurology, Faculdade de Medicina FMUSP, Universidade de São Paulo, São Paulo, Brazil

3. Motor Neuron Disease Research Centre, Faculty of Medicine, Health and Human Sciences, Macquarie Medical School, Sydney, Australia

Supporting material

Figure S1	Schematic overview of the experimental workflow used in this study. Original immunoblot images corresponding to the quantification shown in Figure 2.
Figure S2	Original immunoblot images corresponding to the quantification shown in Figure 3
Figure S3	Original immunoblot images corresponding to the quantification shown in Figure 4 for autophagy markers
Figure S4	Original immunoblot images corresponding to the quantification shown in Figure 5
Table S1	Details of patients included in this study: Diagnostic, recurrence status, gender, age, localization, follow-up, death, Loss Chr.22, and NF2 status
Table S2	Differentially regulated proteins between the comparison of Grade I (GI) and Grade II (GII) groups (p-value < 0.05)
Table S3	Analysis of Gene ontology, domains, and enriched pathways (FDR < 0.05)
Table S4	Limma output file on the transcriptomics data provided by Patel et. al (PMID: 31591222)
Table S5	Protein Groups
