# Supplementary material for: Proteomic Analysis Identifies ATE1-Dependent Arginylation Dysregulation across Meningioma Grades
Source: J Proteome Res. 2026 Jun 29;25(8):3926–36. doi: 10.1021/acs.jproteome.5c01173 (PMC13459535; doi:10.1021/acs.jproteome.5c01173)
Supplement: Supplementary file 2 [file pr5c01173_si_002.pdf]

Supplementary Figures

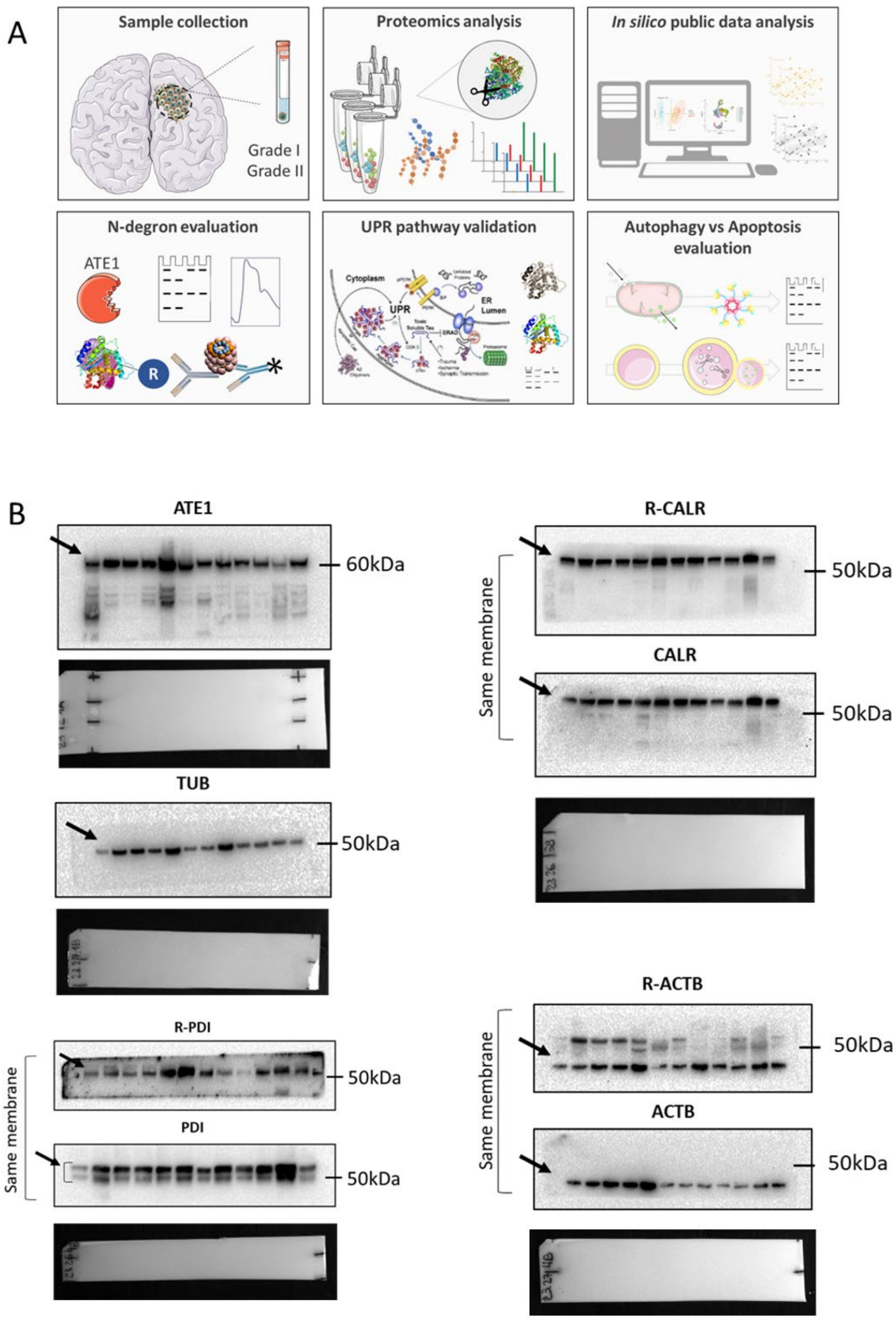

Figure S1. (A) Schematic overview of the experimental workflow used in this study. **(B)** Original immunoblot images corresponding to the quantification shown in Figure 2. Black arrows indicate the bands quantified using ImageJ.

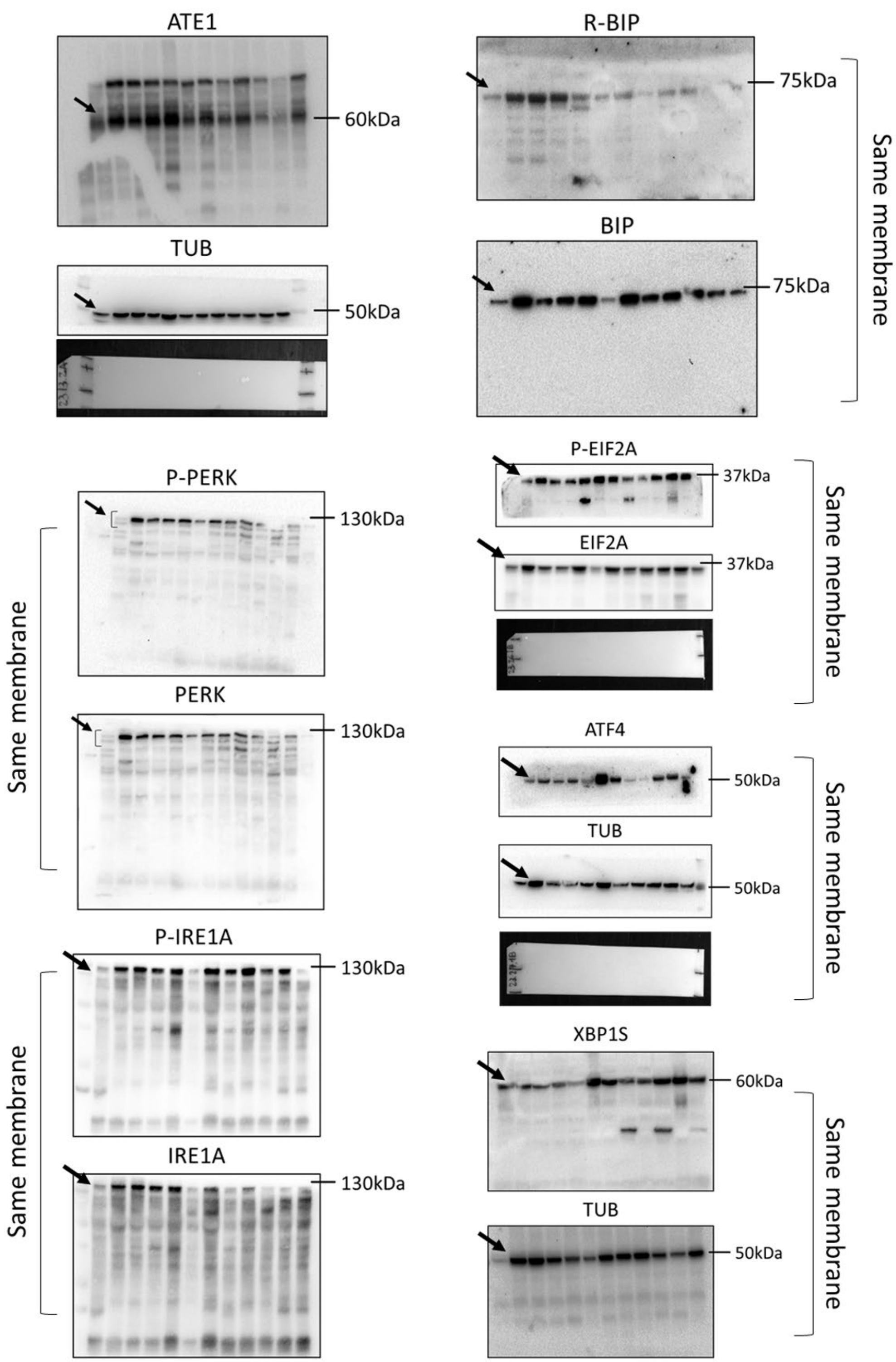

Figure S2. Original immunoblot images corresponding to the quantification shown in Figure 3. Black arrows indicate the bands quantified using ImageJ.

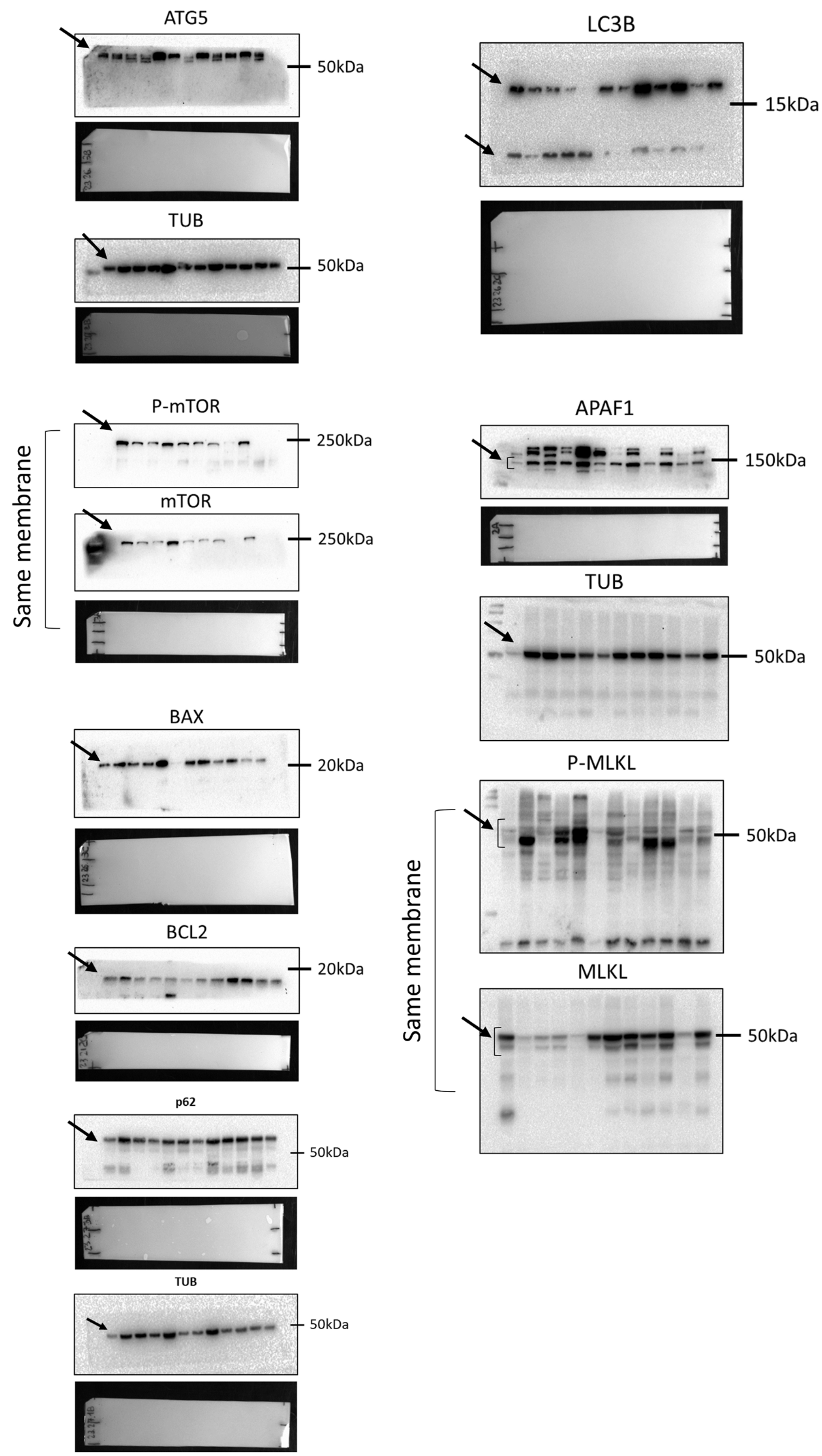

Figure S3. Original immunoblot images corresponding to the quantification shown in Figure 4 for autophagy markers **(A)** and cell death markers **(B).** Black arrows indicate the bands quantified using ImageJ. Both bands were quantified for P-MLKL, MLKL, and APAF1, indicated by square brackets.

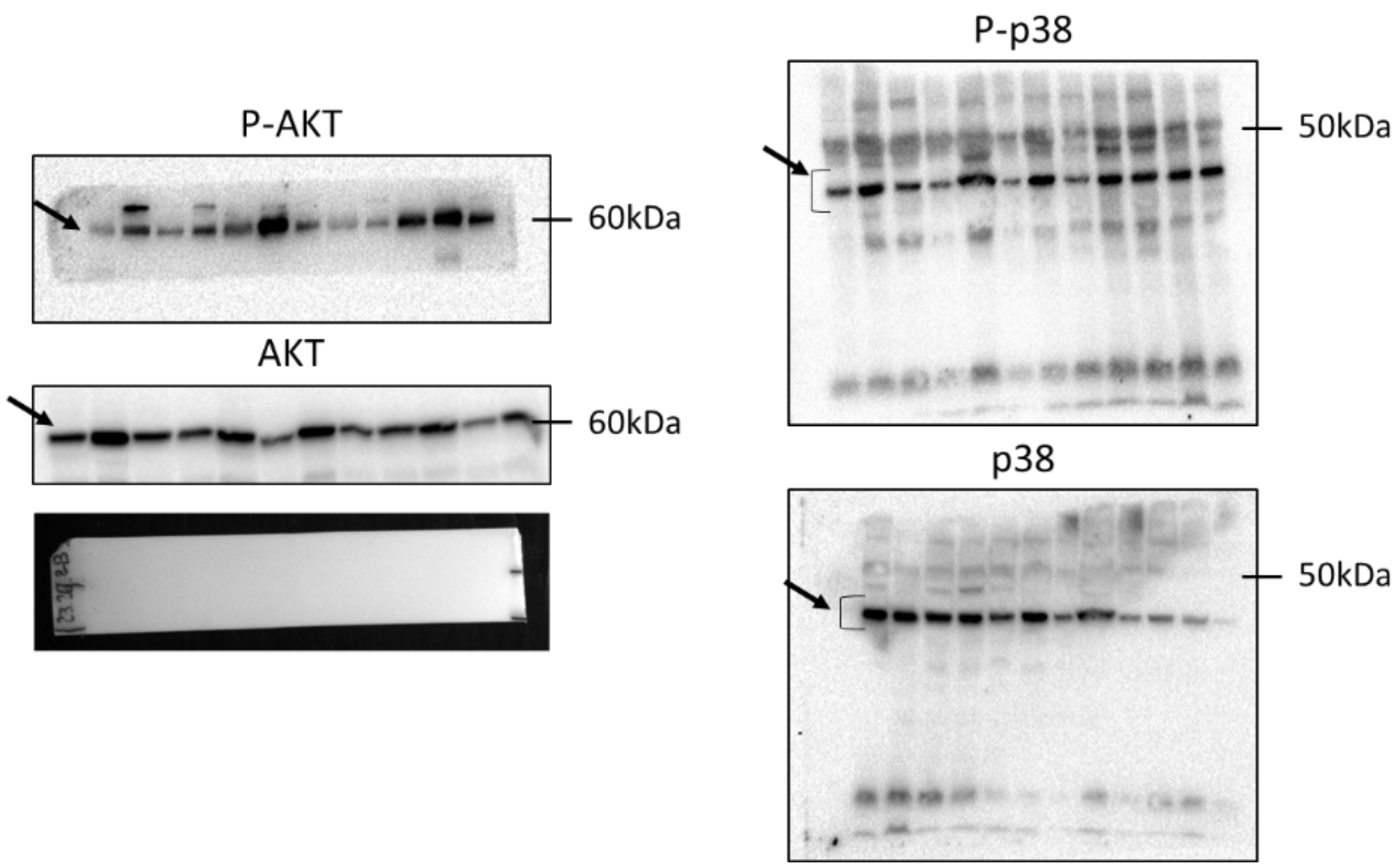

Figure S4. Original immunoblot images corresponding to the quantification shown in Figure 5. Black arrows indicate the bands quantified using ImageJ.
